# Isn’t it time to abandon ARDS? The COVID-19 lesson

**DOI:** 10.1186/s13054-021-03748-6

**Published:** 2021-09-06

**Authors:** L. Gattinoni, J. J. Marini

**Affiliations:** 1 Department of Anesthesiology, Medical University of Göttingen, Robert Koch Straße 40, 37075 Göttingen, Germany; 2 Department of Pulmonary and Critical Care Medicine, University of Minnesota and Regions Hospital, St. Paul, MN USA

In 1967, Ashbaugh et al. [1] reported 12 patients having respiratory failure of different origins who presented a set of similar features. According to current parlance, those elements might constitute a phenotype: (1) hypoxemia refractory to oxygen administration; (2) low respiratory system compliance; (3) bilateral x-ray infiltrates; and for non-survivors, heavy lungs at autopsy. This collection of characteristics, manifesting itself as respiratory difficulty, was called the adult respiratory distress ‘syndrome’ (ARDS) by analogy with similar features observed in premature neonates with surfactant deficiency, termed infant respiratory distress syndrome. (syndrome = symptoms which run together).

The logic behind ‘ARDS’ was that appropriate treatment, primarily of hypoxemia, was similar across patients, regardless of causal etiology (i.e., disease). Some authors [2] questioned the wisdom of lumping together as a unique syndrome such sharply different diseases. The term ‘ARDS’, however, rapidly gained traction and transformed into “Acute” instead of “Adult” Respiratory Distress. ARDS rapidly became the signature “disorder” of the newly fledged discipline of intensive care [3].

Because lungs and clinical manifestations of ‘ARDS’ looked superficially similar regardless of etiology, it was reasoned that the respiratory treatment should be essentially the same. Quite quickly this became the universally accepted approach. One clear advantage of lumping together different diseases into a single entity was that large, randomized trials of treatment became feasible. Indeed, since the number of trial patients required to show significant clinical differences of 5–6% for key variables might require more than two thousand patients, the need to adopt definitions broad enough to allow enrolment of a sufficient number of patients became evident. We believe that this has been a main driver for modifications to the ARDS definition that occurred over subsequent decades. The refractory hypoxemia of Ashbaugh’s original definition of ARDS became first PaO_2_/FiO_2_ ratio 300 mmHg and then 200, and the core feature of low compliance was excluded from later definitions on the questionable basis that measures of respiratory mechanics add little predictive value [4]. Such definitional simplification facilitated study enrollment. For many clinicians, each definitional refinement solidified ‘ARDS’ as a descriptor of a distinct “disease-like” entity. As an example, in a widely quoted paper we find: “the rate of clinician recognition of ARDS was low, with 40% of all cases not being diagnosed” [5]. Which diagnosis? The Ashbaugh? The Chicago? The Berlin? Does it really matter [6]?

Considering ARDS as a ‘disease’ with specific treatment had some advantages; through randomized trials using the broadened ARDS definition, we confirmed that gentle treatment of the lung is the most efficacious approach to this respiratory syndrome. We should not forget, however, that all knowledge from randomized trials has derived primarily from ARDS populations with bacterial pneumonia and septic abdominal disease [7]. Other causes for ARDS are rather sparsely represented. Meanwhile, physiological and pathophysiological studies dissect differences among ARDS populations with distinguishable characteristics and treatment responses, beginning with pulmonary and extra-pulmonary [8]. Indeed, there is now progressive pressure towards ‘personalizing’ medicine, a process which, in a sense, is exactly the opposite from the lumping used to define a syndrome. Against this framework, COVID-19 pneumonia abruptly appeared and changed our lives. Did also it change our thinking?

As many COVID-19 patients present to the hospital with acute bilateral lung opacities the label of “ARDS” has been reflexively assigned and standard treatments for ARDS applied, in line with guidelines of the panels representing the National Institutes of Health and the Surviving Sepsis Campaign [9]. The recommended ‘lung protective’ strategy (low tidal volume and PEEP guided by published PaO_2_/FiO_2_ tables) led to the early use of PEEP levels exceeding 14 cmH_2_O [10]. However, front-line physicians treating these patients soon recognized that regardless of the severity of hypoxemia, the early stage COVID-ARDS lungs were unusually compliant and gas filled. At the same time, the prerequisites for PEEP to work, i.e., lung collapse and recruitability of *functional* units, were marginal, and unlike traditional ARDS, hypoxemia at COVID’s early stage was due primarily to impaired perfusion regulation, rather than true shunt. Moreover, in patients who failed to improve, compliance impressively deteriorated and pathophysiologic mechanisms dramatically changed. These findings introduced a question that initiated hot debate: Are treatment guidelines derived from studies of patients with ARDS caused by bacterial pneumonia and sepsis (which are characterized by recruitable ‘baby’ lungs) well suited to COVID-caused ARDS distinguished by unrecruitable and mechanically evolving ‘adult’ lungs? The answer is important; respiratory treatment likely plays a key role in determining outcome, as the reported ICU mortality rates for the same disease significantly varied between different ICUs [11, 12].

COVID-19 has clearly taught us that this “atypical” form of ARDS requires different treatment than “typical” ARDS. Similar reasoning could be applied for the pulmonary versus extra-pulmonary or for the inflammatory versus non-inflammatory phenotypes. This progressive migration towards “personalized” medicine implies the loss of the primary therapeutic advantage that initially led to lumping that justifies a uniform approach.

At this stage, two different scenarios are possible to accommodate the discordant and inconvenient COVID-19 observations: (1) modify, rearrange, and rethink the ARDS definition; or (2) abandon the ARDS term as understood in its present form  (Table 1). In support of the first option, it has been proposed to expand the ARDS definition by modifying the radiological criterion to include ultrasound imaging, the gas-exchange criterion to allow pulse oximetry [13], and the oxygenation support criterion to recognize the use of high-flow oxygen and moderate PEEP [14]. It is difficult for us to understand the advantages of this ‘ultra-lumping’ approach [15]. If that course is taken, nearly all forms of respiratory distress will be diagnosed as ‘ARDS’. Why should we do this if appropriate management should be different? It seems more logical simply to label the diseases as they are: for example, pneumococcal respiratory distress, herpes respiratory distress, pancreatitis respiratory distress, etc. This de-lumping’ approach would push our thinking towards truly personalized medicine, realizing that not only the etiological treatment but also the appropriate respiratory approach might well be different in different situations and at different stages of the disease process.

**Table 1:** Keeping and abandoning ARDS: advantages and disadvatages

	Keep and modify ARDS definition	Abandon ARDS definition
Advantages	Easy patient categorization and labeling Facilitated enrollment in RCTs Standardized guidance of treatment	Recognizes need to personalize therapy Focuses on patient-relevant characteristics Encourages best responses to ∆’s over time
Drawbacks	Non-uniformity encourages inappropriate Rx Promotes RCT enrollment of unqualified Pts May inform misleading treatment guidelines	Universal standards for Rx difficult to establish Precision inhibits RCT design and enrollment Often requires mastery of bedside physiology

*RCT* randomized clinical trial, *∆* change, *Rx* treatment, *Pts* patients

## Data Availability

Not applicable.
